# Associations between physical activity and depressive symptoms in women

**DOI:** 10.1186/1479-5868-5-27

**Published:** 2008-05-06

**Authors:** Megan Teychenne, Kylie Ball, Jo Salmon

**Affiliations:** 1 Centre for Physical Activity and Nutrition Research, School of Exercise and Nutrition Sciences, Deakin University, 221 Burwood Hwy, Burwood, VIC, 3125, Australia

## Abstract

**Background:**

The high prevalence of depression in women is an increasing public health concern. Although studies have found associations between physical activity (PA) and depression, little is known about the optimal domain, dose and social context of PA for reducing the risk of depression. This study aimed to investigate associations between specific components of PA (domain, dose and social context) and odds of depressive symptoms in women.

**Methods:**

The sample included 1,501 women, aged 18–65. Analyses were performed using cross-sectional data collected from a mail-out survey in 2004. The survey included self-report measures of PA behaviours and depressive symptoms. Crude and adjusted (age, marital status and physical health) odds ratios (OR) and 95% confidence intervals (CI) were calculated for each component of PA and odds of depressive symptoms using logistic regression analyses.

**Results:**

Those who reported more than 3.5 hours leisure-time PA per week had lower odds of depressive symptoms when compared to those who undertook less than this. No other domains of PA (eg. work-related, transport-related or domestic activity) were associated with odds of depressive symptoms. Odds of depressive symptoms were lower among women who reported more than 1.5 hours of moderate-intensity (OR = 0.67, CI = 0.45–0.98) or more than 1.75 hours vigorous-intensity (OR = 0.60, CI = 0.42–0.84) leisure-time PA per week. Being discouraged to be active by others was associated with higher odds of depressive symptoms (OR = 2.28, CI = 1.00–5.16), whilst being active with a family member was associated with lower odds of depressive symptoms (OR = 0.61, CI = 0.43–0.87).

**Conclusion:**

Acknowledging the cross-sectional design, these findings suggest that the domain and social context of PA may be more important for mental health among women than simply the total dose of PA.

## Introduction

Depression is a major cause of physical and psychosocial illness and all-cause mortality [1]. Depression affects more than 340 million people worldwide [2], with women at particular risk [3]. Conventional management of depression has typically involved treatment from clinical professionals such as general practitioners, psychiatrists, psychologists, and has included counselling and anti-depressant medication [4]. However, in recent years research has focused on the role of physical activity as a potential component in the prevention and/or management of depressive symptoms or clinical depression [1].

Several reviews examining observational and intervention studies have assessed the relationship between physical activity and depression/depressive symptoms [5-7]. These reviews generally draw a similar conclusion: that physical activity is positively associated with reduced likelihood of depression or depressive symptoms. However, little is known about the specific components of physical activity that are important, particularly the dose, domain or social context of physical activity that might confer mental health benefits.

Whilst numerous studies have explored the associations between the dose – particularly the frequency and/or duration of physical activity – and likelihood of depression, findings from these studies were varied. A number of observational [8-10] and intervention studies [11,12] concluded that higher levels of physical activity (eg. greater than 60 minutes of physical activity per week) were associated with lower odds of depression. In contrast, other studies [13-16] have found that even low levels of physical activity (eg. exercising as little as 30 minutes per week) was related to improved mental health. Another study [11] has concluded specifically that the commonly recommended 'public health dose' of physical activity (at least 30 minutes of moderate intensity physical activity on most, if not all days of the week) [17,18] was an effective treatment for reducing depressive symptoms.

Conflicting results were also found in studies investigating the association between physical activity intensity and depression. Numerous longitudinal [9], cross-sectional [19] and intervention studies [20,21] have found that participating in physical activity at a moderate-intensity was significantly associated with lower odds of depression whilst few studies have examined the relative strengths of associations between physical activity of moderate or high intensity and risk of depression [22,23].

Most studies supporting an inverse relationship between physical activity and risk of depression have focussed on leisure-time physical activity. Very little research has investigated the association of depressive symptoms with other domains of activity, such as work-related, transport-related or domestic physical activity. Only three studies have been identified which specifically compared leisure-time physical activity with physical activity in other domains [24-26], with all three suggesting leisure-time physical activity was more strongly associated with lower odds of depression than physical activity undertaken in any other domain (domestic, transport-related, work). To our knowledge, no previous research has compared leisure-time, domestic, transport-related and work-related physical activity with odds of depressive symptoms in the same study.

It has been hypothesized that the associations between physical activity and depressive symptoms may be due to the social support and interaction that comes with physical activity undertaken in a group or social setting [27]. Little research, however, has examined this hypothesis. Only one intervention study specifically compared physical activity undertaken alone with that undertaken with others [22]. It found that physical activity had a moderate effect on alleviating depressive symptoms regardless of the social context in which it was undertaken.

The aim of this study was to examine the associations between various components of physical activity (dose, domain and social context) and odds of depressive symptoms in women using data from a large population-based cohort of women. Due to the cross-sectional nature of this study, only participants' current levels of physical activity and depressive symptoms were examined. Since a dose-response relationship has been previously reported [11], it was hypothesized that higher doses of physical activity (similar to that of the public health recommendations) would be more strongly inversely associated with odds of depressive symptoms than would lower doses. It was also hypothesized that leisure-time physical activity would be more strongly inversely associated with odds of depressive symptoms than other domains of physical activity (eg. work-related, transport-related and domestic), since activities performed during leisure-time are generally more enjoyable and undertaken by personal choice. A third hypothesis was that social physical activities and social support for physical activity would be more strongly associated with lower odds of depressive symptoms than physical activity performed alone or without social support.

## Methods

The analyses in this study were based on cross-sectional survey data collected formerly from 1,554 women aged between 18 and 65. Full details of the methods are described elsewhere [28,29].

### Participants

Participants were recruited from 45 Melbourne suburbs with varying levels of socioeconomic disadvantage, which was based on the Australian Bureau of Statistics SEIFA – Socioeconomic Index for Areas [30]. A total of 15 suburbs of low SEIFA, 15 suburbs of mid SEIFA and 15 of high SEIFA (taken from the lowest septile, middle septile and highest septile) were randomly selected and the electoral roll was then used to randomly select women between the ages of 18 and 65 years living in those areas. There was a slight over sampling of women from low and mid socioeconomic status relative to high (ratio 1.5: 1.2: 1) to counter low response rates typically observed in low socioeconomic status groups [30].

Two separate samples of women were randomly sent either a physical activity survey (n = 2,400) or a healthy eating survey (n = 2,400) and those who responded were given the opportunity to complete the alternative survey. Of those who were sent the physical activity survey, 1,045 women responded initially. Of those who were sent the healthy eating survey initially, 509 women also completed the physical activity survey. Of the resulting sample of 1554 women, 53 (3%) were excluded from analyses because they reported being pregnant (or did not indicate they were not pregnant). This is due to the fact that pregnancy is likely to strongly affect both physical activity levels [31,32] and risk of depression [33]. This left a total of 1501 women whose data were included in the analyses.

### Procedures

The study was approved by the Deakin University Human Research Committee and was performed in compliance with the Helsinki Declaration. Women selected for the surveys received a letter in the mail, informing them that they had been selected to take part in a study on women's health behaviours and that the survey would soon be sent to them. Surveys were posted out to the selected women one week later. Following the Dilman protocol [34], non-respondents received a mailed reminder within three weeks and a second reminder with a replacement survey package a further three weeks later. All respondents provided their informed consent to participate in the study.

### Measures

#### Physical activity

Physical activity was measured using the self-completion long form of the International Physical Activity Questionnaire (IPAQ-L), a reliable and well-validated measure involving a seven-day recall of physical activity [35,36]. Questions included the frequency and duration of time spent undertaking various intensities (walking, moderate and vigorous) of physical activity in leisure-time, transport-related activity, work-related activity and household physical activity. For each of these, participants were required to estimate the frequency (total number of days), and duration (hours and minutes) on one of those days, that they spent undertaking such activities in the past week. Questions included; 'During the last 7 days, on how many days did you do moderate activities (like washing windows, scrubbing floors) inside your home?' and 'How much time did you usually spend on one of those days doing moderate physical activities inside your home?'

Total duration of physical activity was then calculated by multiplying the frequency of activities by the duration within each domain (leisure-time physical activity, domestic physical activity, work-related physical activity, transport-related physical activity). Leisure-time physical activity and work-related physical activity were also summed across intensities (walking, moderate-intensity, vigorous-intensity). All physical activity variables were then transformed into categorical variables with three levels (tertiles). Those who reported being unemployed were excluded from the work-related physical activity analyses. Domestic (moderate-intensity only) and transport-related (walking and bicycling) physical activity was not summed across intensities as the questionnaire did not explicitly examine all three intensities in those domains.

Total weekly physical activity was calculated by summing the relevant durations across domains within each intensity of physical activity. This included: total walking (formed from leisure-time physical activity, work and transport-related physical activity domains); total moderate-intensity (formed from leisure-time physical activity, work and domestic physical activity domains); and total vigorous-intensity (formed from leisure-time physical activity and work-related physical activity domains). Total overall weekly dose of physical activity across all domains was also calculated. All continuous measures of physical activity were transformed into tertile categories.

#### Social context

Social support for physical activity was measured using items adapted from published and validated scales [37-40]. Participants were asked to report on a five-point scale ranging from 'never' to 'very often' (subsequently collapsed into three categories: never/rarely, sometimes, or often), how frequently they participated in physical activity with family and friends/colleagues in the past year [38]; whether others discouraged them from physical activity [39]; and whether they had someone to exercise with [39]. Participants were asked to report using a five-point scale ranging from 'strongly disagree' to 'strongly agree' (subsequently collapsed into two categories: strongly agree/agree, vs. all other responses) whether they had someone to exercise with in their neighbourhood [37], whether they were a member of a sporting/recreational club (yes/no) [40], as well as whether they had a dog which they walked regularly (yes/no).

#### Depressive symptoms

Mental health characteristics of participants were measured using the 30-item version of the General Health Questionnaire [41]. This includes questions relating to symptoms of depression as indicators of risk of poor mental health. Questions were based on whether depressive symptoms had been present recently (in the 'last couple of weeks'). The measurement properties of this tool have been widely reported and it has been found to provide an accurate prediction of those at risk of depression [42]. Furthermore, the General Health Questionnaire has been shown to be associated with relation to physical activity in previous cross-sectional studies [13,43]. In this study, total GHQ-30 scores greater than four were used to indicate that the participant was currently experiencing depressive symptoms [44].

### Covariates

Age (categorized as under 30; 30–39; 40–49; or 50 years or over), marital status (married/defacto; separated/widowed/divorced; or never married) and physical health (whether or not respondents reported the presence of a long-term illness or disability that prevents them from being physically active) were included in the analyses as potentially confounding factors, since these variables were found to be bivariately associated with the presence of depressive symptoms in chi-square analysis. Other possible confounders including education, employment status and having children were not associated with the presence of depressive symptoms and were therefore not included as covariates.

### Statistical Analyses

Analyses were conducted using the SPSS version 12.0 statistical software package. Univariate analyses were performed in order to describe the demographic characteristics of participants, their participation in different intensities and domains of physical activity, social characteristics of physical activity, and presence of depressive symptoms. Crude and adjusted (controlling for age, marital status and physical health) odds ratios (OR) and 95% confidence intervals (CI) were then calculated for each of the physical activity variables and presence of depressive symptoms using logistic regression analyses.

## Results

Table 1 presents the socio-demographic characteristics and presence of depressive symptoms among participants. The mean age of participants was 42 years. The majority of the women were born in Australia (75%) and were married or in a defacto relationship (65%). A total of 615 (41%) participants reported their highest qualification as completing year 12 (or equivalent) or an apprenticeship or certificate/diploma, with 566 (37%) having completed a university or higher degree. Over one-quarter of the women were employed in a professional occupation (27%) and the majority of women surveyed did not have children living at home (60%). These demographic characteristics are comparable to those reported in other population-based studies conducted in Melbourne and nationally [45-48]. A total of 421 (30%) of participants were classified as currently experiencing depressive symptoms (according to the GHQ-30). This is not dissimilar to previous findings which reported that almost 24% of Australian women had been diagnosed with depression [49].

**Table 1:** Characteristics of participants

**Characteristic**	**N**	**Percent**
***Age***		
29 yrs and under	309	21
30 to 39 yrs	382	25
40 to 49 yrs	358	24
Over 50 yrs	462	31
		
***Country of birth***		
Australia	1142	75
UK	63	4
Italy	23	2
Greece	24	2
New Zealand	9	1
Vietnam	30	2
Other	239	16
		
***Marital Status***		
Married or defacto	979	65
Separated widowed or divorced	203	13
Never married	339	22
		
***Currently have an illness/long-term disability***		
Yes	227	15
No	1260	85
		
***Highest Qualification***		
No formal or up to year 10	335	22
Year 12/trade apprentice/Certificate diploma	615	41
University or Higher degree	566	37
		
***Main Occupation***		
Manager or administrator	119	8
Professional	397	27
Associate professional	123	8
Tradesperson or related worker	63	4
Advanced clerical or service worker	165	11
Intermediate clerical, sales or service worker	157	11
Intermediate production or transport worker	12	1
Elementary clerical, sales or service worker	54	4
Labourer or related worker	75	5
No paid job	235	16
Student	87	6
		
***Children living at home (up to 18 yrs)***		
Yes	554	40
No	828	60
		
***Depressive symptoms***		
No (<= 4)	999	70
Yes (>4)	421	30

Table 2 shows the proportion of women experiencing depressive symptoms according to sociodemographic and physical activity variables from chi-square analyses. The only domain of physical activity to be inversely associated with the presence of depressive symptoms was leisure-time (p = 0.009). Women who reported walking or moderate-intensity physical activity in leisure time were less likely to be experiencing depressive symptoms (p = 0.027 and p = 0.048 respectively). For example, 22% of those who reported more than 1.5 hours of moderate-intensity leisure-time physical activity (i.e. the highest tertile) were currently experiencing depressive symptoms, compared with 31% of those reporting no moderate-intensity leisure-time physical activity.

**Table 2:** Proportion of women experiencing depressive symptoms by sociodemographic characteristics, physical activity dose, domain and social factors

**Variable**	**Category**	**n**	**No depressive symptoms (%)**	**Depressive symptoms (%)**	**P value**
**Age group**	<30 years	286	59	41	0.000
	30–39	342	68	32	
	40–49	316	70	30	
	>50 years	415	80	20	
					
**Marital status**	Married/defacto	872	74	26	0.002
	Separated/widowed/divorced	179	65	35	
	Never married	316	64	36	
					
**Physical health**	No illness/disability	1168	72	28	0.001
	Long-term illness/disability	205	60	40	
					
**LTPA**					
*Walking*	No walking	470	66	34	0.027
	0.1–1.5 hours p/week	396	70	30	
	More than 1.5 hours p/week	498	74	26	
					
*Moderate*	No moderate LTPA	985	69	31	0.048
	0.1–1.5 hours p/week	194	68	32	
	More than 1.5 hours p/week	181	78	22	
					
*Vigorous*	No vigorous LTPA	937	68	32	0.069
	0.1–1.75 hours p/week	185	71	29	
	More than 1.75 hours p/week	239	76	24	
					
*Total LTPA*	Less than 0.67 hours p/week	430	65	35	0.009
	0.67–3.5 hours p/week	461	72	28	
	More than 3.5 hours p/week	449	75	25	
					
**Work PA**					
*Walking*	No walking	424	71	29	0.906
	0.01–3.5 hours p/week	294	70	30	
	More than 3.5 hours p/week	288	69	31	
					
*Moderate*	No moderate work PA	488	71	29	0.955
	0.1–2.67 hours p/week	257	70	30	
	More than 2.67 hours p/week	261	70	30	
					
*Vigorous*	No vigorous work PA	652	72	28	0.342
	0.1–3 hours p/week	198	68	32	
	More than 3 hours p/week	168	67	33	
					
*Total Work PA*	less than 0.28 hours p/week	315	73	27	0.538
	0.28–7.33 hours p/week	333	70	30	
	more than 7.33 hours p/week	330	70	31	
					
**Transport PA**	Less than 0.58 hours p/week	436	69	31	0.698
	0.58–2.33 hours p/week	436	72	28	
	More than 2.33 hours p/week	462	70	30	
					
**Domestic PA**	Less than 1.93 hours p/week	437	68	32	0.507
	1.93–6 hours p/week	480	72	28	
	More than 6 hours p/week	452	71	29	
					
**Total weekly PA**					
*Walking*	Less than 3 hours	364	70	30	0.841
	3.1–9 hours	313	70	30	
	More than 9 hours	309	71	29	
					
*Moderate*	Less than 3 hours	355	68	32	0.301
	3.1–10 hours	320	70	30	
	More than 10 hours	319	73	27	
					
*Vigorous*	No vigorous PA	424	71	29	0.905
	0.1–4 hours	365	70	30	
	More than 4 hours	215	71	29	
					
*Total overall*	Less than 8.5 hours	322	68	32	0.391
	8.5–23 hours	307	73	27	
	More than 23 hours	315	70	30	
					
**Social Factor**					
*Others discourage me from physical activity*	Never/rarely	1138	72	28	0.002
	Sometimes	171	61	39	
	Often	27	52	48	
					
*Have no one to exercise with*	Never/rarely	625	75	25	0.037
	Sometimes	372	71	29	
	Often	175	66	34	
					
*Member of sporting club*	No	980	69	31	0.044
	Yes	378	74	26	
					
*How often a family member is active with you*	Never/rarely	519	65	35	0.011
	A few times	444	72	28	
	Often	253	74	26	
					
*How often a friend is active with you*	Never/rarely	755	69	31	0.285
	A few times	294	67	33	
	Often	206	73	27	
					
*Have a dog you walk regularly*	No	989	70	30	0.578
	Yes	373	71	29	
					
*Have someone to walk with in the neighbourhood*	Agree	555	74	26	0.037
	Other (than agree)	803	68	32	

PA, physical activity; LTPA, leisure-time physical activity

Being discouraged from physical activity and having no one to exercise with were associated with an increased presence of depressive symptoms (p = 0.002 and p = 0.037 respectively), whilst being a member of a sporting/recreational club, having someone to walk with in the neighbourhood and being active with a family member were associated with a lower presence of depressive symptoms (p = 0.044, p = 0.037, and p = 0.011 respectively).

Table 3 shows the crude and adjusted OR and 95% CI from logistic regression models predicting odds of depressive symptoms according to physical activity variables. Results from the unadjusted logistic regression models predicting odds of depressive symptoms according to duration of physical activity (summed across intensities) within each domain, showed that compared to those in the lowest tertile of leisure-time physical activity (i.e. <0.67 hours/week leisure-time physical activity), those women in the highest tertile (>3.5 hours/week) had lower odds of depressive symptoms (p = 0.002). Adjusting for covariates had little effect on this association. No other physical activity domain was associated with odds of depressive symptoms in logistic regression models.

**Table 3:** Crude and adjusted^a ^odds of depressive symptoms according to physical activity variables (hrs/wk)

**Variable**	**Category (p/week)**	**Crude odds ratio**	**95% CI**	**P value**	**Adjusted^a ^odds ratio**	**Adjusted^a ^95% CI**	**P value**
**LTPA**							
*Walking*	No walking	1.00			1.00		
	0.1–1.5 hours	0.83	0.62–1.12	0.211	0.87	0.646–1.18	0.370
	More than 1.5 hours	0.68	0.52–0.90	0.007	0.76	0.570–1.01	0.061
							
*Moderate*	No moderate PA	1.00			1.00		
	0.1–1.5 hours	1.05	0.76–1.47	0.763	1.01	0.71–1.42	0.975
	More than 1.5 hours	0.64	0.44–0.93	0.018	0.67	0.45–0.98	0.039
							
*Vigorous*	No vigorous PA	1.00			1.00		
	0.1–1.75 hours	0.90	0.64–1.27	0.538	0.80	0.56–1.16	0.237
	More than 1.75 hours	0.68	0.50–0.95	0.022	0.60	0.42–0.84	0.003
							
*Total LTPA*	Less than 0.67 hours	1.00			1.00		
	0.67–3.5 hours	0.83	0.63–1.10	0.196	0.82	0.61–1.10	0.192
	More than 3.5 hours	0.63	0.47–0.85	0.002	0.64	0.47–0.86	0.004
							
**Work PA**							
*Walking*	No walking	1.00			1.00		
	0.1–3.5 hours	1.03	0.74–1.43	0.866	0.91	0.65–1.28	0.583
	More than 3.5 hours	1.08	0.78–1.49	0.658	0.96	0.69–1.35	0.825
							
*Moderate*	No moderate PA	1.00			1.00		
	0.1–2.67 hours	1.05	0.76–1.46	0.766	0.97	0.67–1.37	0.854
	More than 2.67 hours	1.03	0.74–1.43	0.868	0.95	0.67–1.34	0.762
							
*Vigorous*	No vigorous PA	1.00			1.00		
	0.1–3 hours	1.21	0.85–1.70	0.289	1.11	0.78–1.59	0.569
	More than 3 hours	1.26	0.87–1.81	0.219	1.07	0.73–1.58	0.732
							
*Total work PA*	Less than 0.28 hours	1.00			1.00		
	0.28–7.33 hours	0.83	0.59–1.16	0.276	1.03	0.73–1.47	0.858
	More than 7.33 hours	0.95	0.68–1.32	0.741	1.04	0.73–1.48	0.834
							
**Transport PA**	Less than 0.58 hours	1.00			1.00		
	0.58–2.33 hours	0.89	0.66–1.19	0.414	0.81	0.60–1.10	0.181
	More than 2.33 hours	0.97	0.73–1.29	0.833	0.90	0.67–1.21	0.509
							
**Domestic PA**	Less than 1.93 hours	1.00			1.00		
	1.93–6 hours	0.85	0.64–1.13	0.253	0.92	0.68–1.23	0.560
	More than 6 hours	0.90	0.67–1.19	0.441	1.05	0.77–1.42	0.783

**Total weekly PA**							
*Walking*	Less than 3 hours	1.00					
	3.1–9 hours	0.98	0.70–1.36	0.896	0.93	0.66–1.31	0.682
	More than 9 hours	0.91	0.65–1.27	0.568	0.83	0.59–1.17	0.285
							
*Moderate*	Less than 3 hours	1.00					
	3.1–10 hours	0.87	0.63–1.20	0.397	0.90	0.63–1.26	0.532
	More than 10 hours	0.77	0.55–1.07	0.124	0.79	0.56–1.12	0.184
							
*Vigorous*	No vigorous PA	1.00					
	0.1–4 hours	1.06	0.78–1.44	0.721	0.94	0.68–1.30	0.714
	More than 4 hours	0.98	0.68–1.41	0.915	0.79	0.54–1.17	0.237
							
*Total overall*	Less than 8.5 hours	1.00					
	8.5–23 hours	0.79	0.56–1.11	0.174	0.78	0.54–1.11	0.164
	More than 23 hours	0.92	0.66–1.28	0.618	0.90	0.63–1.27	0.543

^a^Adjusted for age, marital status and physical health. PA, physical activity; LTPA, leisure-time physical activity

When examined according to specific physical activity intensities, the unadjusted results showed that compared to those who reported no walking in leisure time, those in the highest walking tertile (>1.5 hours/week) had lower odds of depressive symptoms (p = 0.007). However, this association was only marginally significant when adjusting for confounders. Compared to those who reported no moderate leisure-time physical activity, adjusted results showed that those in the highest tertile (>1.5 hours/week) had lower odds of depressive symptoms (p = 0.039). Similarly, compared with those who reported no vigorous leisure-time physical activity, those in the highest tertile (more than 1.75 hours/week) had lower odds of depressive symptoms (p = 0.003).

There were no statistically significant associations between work-related physical activity of any intensity, and odds of depressive symptoms, in either unadjusted or adjusted logistic regression models. There were also no associations between total walking, moderate, vigorous or overall physical activity (summed across all domains) and odds of depressive symptoms.

Table 4 shows the crude and adjusted OR and 95% CI from logistic regression models predicting odds of depressive symptoms according to the social context of physical activity. The unadjusted results indicate that compared to those who reported never/rarely being discouraged from physical activity, those who reported sometimes being discouraged had higher odds of depressive symptoms (OR = 1.6; p = 0.005), while those who reported often being discouraged had even higher odds of depressive symptoms (OR = 2.4; p = 0.025).

**Table 4:** Crude and adjusted^a ^odds of depressive symptoms according to the social context of physical activity.

**Social factor**	**Category**	**Crude odds ratio**	**95% CI**	**P**	**Adjusted^a ^odds ratio**	**Adjusted^a ^95% CI**	**P**
**Others discourage me**	Never/rarely	1.00			1.00		
	Sometimes	1.62	1.16–2.26	0.005	1.70	1.20–2.42	0.003
	Often	2.39	1.11–5.15	0.025	2.28	1.00–5.16	0.049
							
**No one to exercise with**	Never/rarely	1.00			1.00		
	Sometimes	1.25	0.94–1.66	0.134	1.16	0.86–1.56	0.333
	Often	1.57	1.09–2.25	0.014	1.37	0.94–1.99	0.107
							
**Member of a sporting club**	No	1.00			1.00		
	Yes	0.76	0.58–0.99	0.045	0.76	0.57–1.01	0.061
							
**How often a family member is active with you**							
	Never/rarely	1.00			1.00		
	A few times	0.73	0.55–0.96	0.022	0.77	0.58–1.03	0.074
	Often	0.64	0.46–0.90	0.009	0.61	0.43–0.87	0.007
							
**How often a friend is active with you**	Never/rarely	1.00			1.00		
	A few times	1.12	0.84–1.49	0.439	1.06	0.79–1.44	0.684
	Often	0.82	0.58–1.15	0.248	0.83	0.58–1.19	0.315
							
**Have a dog you walk regularly**	No	1.00			1.00		
	Yes	0.93	0.71–1.20	0.578	0.94	0.71–1.23	0.633
							
**Have someone to walk with in your neighbourhood**	Agree	1.00			1.00		
	Other (than agree)	0.77	0.61–0.98	0.037	0.80	0.62–1.03	0.083

^a^Adjusted for age, marital status and physical health.

These associations were only slightly altered (to OR = 1.7 and 2.3 respectively) in the adjusted analyses. Similarly in the unadjusted models, compared with those who reported being rarely/never active with a family member, those who reported being active a few times per year with a family member had lower odds of depressive symptoms (OR = 0.73; p = 0.022), while those who reported often being active with a family member had even lower odds of depressive symptoms (OR = 0.64; p = 0.009). These associations also changed only marginally (to OR = 0.77 and 0.61 respectively) after adjusting for covariates.

Compared to those who reported never/rarely having no-one to exercise with, the unadjusted results showed that those who often have no-one to exercise with had higher odds of depressive symptoms (p = 0.014). Although the adjusted results showed a similar trend, this was no longer statistically significant after covariates were included in the model. Being a member of a sporting club was significantly associated with lower odds of depressive symptoms before adjusting for confounders; however, this association only approached significance in the adjusted model (p = 0.061).

No association was evident between the frequency of having a friend/colleague to exercise with and odds of depressive symptoms, or having a dog to walk regularly and odds of depressive symptoms in either unadjusted or adjusted logistic regression analyses. Similarly, the adjusted results indicate that those who agreed that they have someone to walk with in the neighbourhood had lower odds of depressive symptoms, but this was not statistically significant (p = 0.083).

## Discussion

The hypothesized associations between overall physical activity dose and odds of depressive symptoms were not found in this study, as there were no significant associations between total walking, moderate, vigorous or overall physical activity (i.e. across all domains) and odds of depressive symptoms. These findings imply that it may not be the physiological effects of physical activity that are important in reducing the odds of depressive symptoms, since, if this were the case, a stronger association of odds of depressive symptoms with total dose (across all domains) of physical activity would have been expected.

The only domain of physical activity found to be inversely associated with odds of depressive symptoms was leisure-time activity. When controlling for age, marital status and physical health, those who reported more than 31/2 hours of total leisure time physical activity per week (summed across intensities) had lower odds of depressive symptoms when compared to those who undertook less than this. In fact, no significant associations were found between undertaking less than 3 1/2 hours of total weekly leisure-time physical activity and odds of depressive symptoms, suggesting that a high duration of leisure-time physical activity may confer greater mental health benefits. This finding supports the dose of physical activity recommended in the US, UK and Australian national physical activity guidelines which suggest a minimum duration of physical activity equivalent to 2 1/2 to 3 1/2 hours per week (at least 30 minutes on most, if not all days of the week [17,18,50]. This is consistent with findings from several observational [8,51] and intervention studies [52,53] which found the public health dose of exercise to be strongly associated with a lower risk of depression. The Australian national physical activity guidelines are based largely on evidence concerning benefits for physical health. These findings suggest they could be extended to mental health. Although other studies have also found a shorter duration of physical activity to be associated with lower risk of depression [14,19,54], these studies used measures of physical activity and risk of depression that differed from the current study. For example, unlike the current study, the method used by Hassmen et al [19] was unable to determine different intensities or domains of physical activity.

When examining different intensities of leisure-time physical activity, the results showed reduced odds of depressive symptoms amongst women in the highest tertiles of moderate- and vigorous-intensity leisure-time physical activity, but those performing vigorous-intensity physical activity showed an even greater reduction in odds. These findings are supported by previous studies that found a strong association between undertaking higher durations of moderate-intensity [9] or vigorous-intensity [51] physical activities and odds of depression. Findings from the current study suggest that the greater the dose (intensity and duration) of leisure-time physical activity, the lower the odds of depressive symptoms. However, there was also an inverse trend approaching significance between depressive symptoms and walking, suggesting that vigorous-intensity physical activity may not be necessary to achieve the reduced odds of depressive symptoms associated with physical activity. Targeting physical activity of specific intensities perhaps guided by personal preference and enjoyment may be a point of consideration when prescribing or recommending a dose of physical activity to confer mental health benefits, as this may result in an increased likelihood of maintenance of the training program and consequently maintenance of reduced depressive symptoms [55]. However, given there were strong inverse associations of depressive symptoms with vigorous activity, women should be encouraged to include at least some activity of this intensity in their weekly physical activity routine if possible.

Several researchers have hypothesised that physical activity reduces risk of depression through physiological pathways – for instance, exercise may activate endorphin secretion, which reduces pain and produces a euphoric sensation [1]. However, the finding that leisure-time physical activity was the only domain associated with odds of depressive symptoms suggests some alternative possible explanations, such as a sense of enjoyment or a perceived control or choice when undertaking activities in leisure-time that may be lacking when physical activity is undertaken in domains other than leisure-time (i.e. work-related, domestic, and transport-related). Alternatively, the finding that different domains of physical activity were differentially associated with odds of depression could be attributed to recall difficulties, as leisure-time physical activity may be more accurately recalled than physical activity undertaken in other domains [56]. This finding is in contrast to previous observational studies [24,25] that did find weak correlations between physical activity undertaken in other domains (domestic and transport) and likelihood of depression. However, it was difficult to interpret the results from those studies in terms of optimal domain of physical activity for reducing likelihood of depression, since in these studies, domain was simply equated with intensity (eg. domestic physical activity = low-intensity physical activity), yet physical activities undertaken from the domestic domain are not always of a low intensity, and not all low-intensity physical activity is necessarily domestic in nature [57]. Few studies have examined the association between multiple domains of physical activity and likelihood of depression. However, concurrent with findings in this study, one previous observational study that compared leisure-time physical activity with domestic and work-related physical activity, found only leisure-time physical activity to be inversely associated with depression [26].

The findings of the current study are generally consistent with the social interaction hypothesis, which posits that the improvements in mental health following exercise are at least partly related to the mutual support and social relationships that are provided when participating in physical activity [27]. In the current study, lower social support for physical activity (eg. being discouraged by others) was associated with higher odds of depressive symptoms, whilst greater social support (eg. being often physically active with a family member) was associated with lower odds of depressive symptoms. These findings, however, are not consistent with one previous study of men and women that suggested that physical activity was associated with lower odds of depressive symptoms regardless of the social format [22]. The sample in that study differed from the current study (i.e. included both men and women), which may explain the contrasting results; women may value and benefit more from the social aspects of physical activity more than men [58]. In the present study, no association was seen between being physically active with a friend/colleague, having no-one to exercise with or having a dog to walk regularly and odds of depressive symptoms. It may be that the support for being physically active from family is more important for mental health among women than support from other sources.

Several limitations of this study should be acknowledged. Firstly, the cross-sectional nature of the study did not allow us to determine the causality or direction of relationships. For example, those without depressive symptoms may be more capable and motivated to participate in physical activity. We were also unable to determine whether the association between support for physical activity and depressive symptoms is due to the social aspect of the physical activity, or due to having a generally more supportive social network. Further longitudinal and intervention studies are needed to confirm the associations observed here, and also to investigate the mechanisms by which physical activity might protect against risk of depression. Such hypothesized mechanisms include 'distraction', whereby improvements in mental well-being following exercise are due to diverting negative thoughts and unpleasant stimuli during the activity [1]; or 'mastery', whereby improvements following physical activity are due to achieving goals and a feeling of success [1]. This study relied on self-report measures which possess the potential for error in judgement, recall difficulties and the possibility of socially desirable responses. Nevertheless, measures of depressive symptoms, physical activity and social influences were valid and widely accepted [35,37-40,42]. Finally, the response rate for the physical activity survey was only 43.5%, whilst the response rate from those who received the diet questionnaire first was 21.2%. This is a potential limitation of our study as participants who responded may be more interested in physical activity and possibly be more active than non-respondents. However, comparison of physical activity levels of the women in this study with those of Australian women reported elsewhere [46] showed similar levels of activity across the two samples.

Strengths of this study include the large, population-based sample of participants who were generally representative of Australian women in terms of both their physical activity levels and depressive symptoms [46,49]. Further, to our knowledge, no previous research has compared leisure-time, domestic, transport-related and work-related physical activity with odds of depressive symptoms in the same study, and few have examined the social context of physical activity and its association with odds of depressive symptoms. Therefore, this study provides a novel perspective on the optimal dose, domain and social context of physical activity that may confer mental health benefits in women.

## Conclusion

In summary, acknowledging the need for confirmation of causal effects, the results from this study suggest that promoting a relatively high duration of leisure-time physical activity could be an important strategy in the prevention of depressive symptoms in women, with additional benefits potentially resulting at higher intensities. Furthermore, programs aimed at reducing the odds of depressive symptoms through the promotion of physical activity could consider the potential for additional benefits associated with physical activity undertaken in a social context by developing strategies emphasizing social aspects of leisure-time physical activity, such as walking groups or exercise classes.

## Competing interests

The authors declare that they have no competing interests.

## Authors' contributions

MT performed the analyses and led the writing of the manuscript. KB conceived of the study, participated in the design, the survey development and helped to draft the manuscript. JS contributed to the survey questionnaire and helped to draft the manuscript. All authors read and approved the final manuscript.
